# Current and Future Patterns of Global Marine Mammal Biodiversity

**DOI:** 10.1371/journal.pone.0019653

**Published:** 2011-05-23

**Authors:** Kristin Kaschner, Derek P. Tittensor, Jonathan Ready, Tim Gerrodette, Boris Worm

**Affiliations:** 1 Evolutionary Biology and Ecology Lab, Institute of Zoology, Albert-Ludwigs-University, Freiburg, Germany; 2 Department of Biology, Dalhousie University, Halifax, Nova Scotia, Canada; 3 Institute of Estuarine and Coastal Studies, Universidade Federal do Pará – Campus de Bragança, Bragança, Pará, Brazil; 4 Protected Resources Division, Southwest Fisheries Science Center, National Marine Fisheries Service, La Jolla, California, United States of America; National Oceanic and Atmospheric Administration/National Marine Fisheries Service/Southwest Fisheries Science Center, United States of America

## Abstract

Quantifying the spatial distribution of taxa is an important prerequisite for the preservation of biodiversity, and can provide a baseline against which to measure the impacts of climate change. Here we analyse patterns of marine mammal species richness based on predictions of global distributional ranges for 115 species, including all extant pinnipeds and cetaceans. We used an environmental suitability model specifically designed to address the paucity of distributional data for many marine mammal species. We generated richness patterns by overlaying predicted distributions for all species; these were then validated against sightings data from dedicated long-term surveys in the Eastern Tropical Pacific, the Northeast Atlantic and the Southern Ocean. Model outputs correlated well with empirically observed patterns of biodiversity in all three survey regions. Marine mammal richness was predicted to be highest in temperate waters of both hemispheres with distinct hotspots around New Zealand, Japan, Baja California, the Galapagos Islands, the Southeast Pacific, and the Southern Ocean. We then applied our model to explore potential changes in biodiversity under future perturbations of environmental conditions. Forward projections of biodiversity using an intermediate Intergovernmental Panel for Climate Change (IPCC) temperature scenario predicted that projected ocean warming and changes in sea ice cover until 2050 may have moderate effects on the spatial patterns of marine mammal richness. Increases in cetacean richness were predicted above 40° latitude in both hemispheres, while decreases in both pinniped and cetacean richness were expected at lower latitudes. Our results show how species distribution models can be applied to explore broad patterns of marine biodiversity worldwide for taxa for which limited distributional data are available.

## Introduction

The global distribution of species diversity and richness has been of interest to naturalists for centuries and remains an important research topic in ecology today [1]. More recently, this quest has been further motivated by systematic conservation planning efforts, which require detailed data on the distribution of biodiversity in space and time [2]. Quantifying patterns of biodiversity can be costly and challenging, particularly in the oceans where most taxa cannot easily be seen and many species are highly mobile with large ranges that extend far into the open oceans [3].

In terms of species number, marine mammals are a relatively small taxonomic group, yet given their biomass and position in the food web they represent an ecologically important part of marine biodiversity [4], [5], [6] Furthermore they are of significant conservation concern, with 23% of species currently threatened by extinction [6]. Therefore, marine mammals often feature prominently in marine conservation planning and protected area design [7], [8], [9]. Their large-scale patterns of biodiversity have only recently been analyzed using expert knowledge [6] or regional observations [10]. Using expert knowledge, Schipper and colleagues [6] delineated the known, or suspected, range of individual species and then overlaid maps to produce global patterns of marine mammal species richness. This approach can accommodate all species on a global scale, but represents a relatively coarse approach that does not distinguish between core and marginal habitats, attributing the same probability of occurrence for a species throughout its range [6]. In addition, resulting patterns remain to be quantitatively validated and cannot be used directly to investigate shifts in distributions under different environmental conditions, since distributions are based on expert knowledge, rather than predictive models that take into account environmental forcings. In contrast, due to the lack of occurrence records for most marine mammal species, existing empirical attempts using sighting surveys to estimate realized cetacean richness have been restricted in taxonomic and spatial coverage, and resulting global predictions may suffer from undersampling [10]. Similar to the trade-offs of different habitat prediction modeling approaches [11], these two methods lie on opposite ends of a spectrum from potentially overpredicting expert-derived (range maps) to potentially underpredicting (empirical sighting surveys) range sizes. Here we present a complementary modelling approach that combines both types of data to make predictions of large-scale marine mammal species distributions using a relative environmental suitability (RES) model [12]; an environmental niche model developed specifically to deal with the prevailing paucity of data for many marine mammal species [12]. The RES model delineates the environmental tolerances of all species with respect to basic parameters known to determine marine mammal distributions directly or indirectly. It does so by combining available data on species occurrence and habitat usage, supplemented by expert knowledge [12]. The relative environmental suitability of different habitats for a given species can then be computed and used to predict long-term mean annual species distributions. Here we superimpose individual species predictions to generate global patterns of species richness, defined as the number of species present in a given area [13], which we subsequently validated using independent survey data.

Bioclimatic envelope models such as the RES models are based on the relationship between species occurrence and environmental proxies, and have been used to explore possible range shifts of marine and terrestrial species under changing environmental conditions [14], [15], although results tend to be sensitive to model assumptions and uncertainties [16], [17], [18]. Global warming is imposing environmental changes on a large scale, and empirical observations indicate shifts in the distributional ranges of many species; these shifts are often consistent with global warming as a driving mechanism [19]. In the oceans, many taxa, ranging from benthic invertebrates to plankton and fish, have shown such range shifts (reviewed in [20]). There is much concern about climate change impacts on marine mammals [21], [22], but the assessment of impacts has mostly been restricted to theoretical considerations [23], [24], [25]. The quantification of possible effects on health [26], food availability [27] and migration [28] remains difficult and impacts are expected to vary for different species [27]. However, species distributions are expected to be affected by temperature and ice cover changes [23], with changes in community structure [29], range expansions into higher-latitude waters [10], [30], and decreases in suitable habitat [31] among the probable outcomes. Here we apply species-specific RES models to explore the possible consequences of temperature change for the global distribution of marine mammal richness in the near future.

## Methods

### Mapping marine mammal richness

We explored marine mammal species richness by overlaying predictions of the relative probability of occurrence for 115 marine mammal species. These included 68 toothed whales (Order: Odontocetii), 15 baleen whales (Mysticetii), and 32 seals and sea lions (Pinnipedia), but excluded all freshwater species, dugongs and manatees (Sirenia), sea otter (*Enhydra lutis*), and polar bear (*Ursus maritimus*). Individual species' ranges were derived from an environmental niche model that predicted distributions and the relative environmental suitability (RES) for different species on a 0.5°x0.5° global grid. Predicted results represent mean annual geographic ranges defined as the maximum area between the known outer-most limits of a species' regular or periodic occurrence [12]. While this definition is inclusive of all areas covered during annual migrations, dispersal of juveniles etc., it specifically excludes extralimital sightings, which are sometimes difficult to distinguish from the core range [32]. The RES modeling approach was developed because of the paucity of marine mammal data available for standard species distribution modelling approaches, and well-known spatial biases in the available data: point occurrence records are currently only available for <60% of known marine mammals [33], and 70% of all available sighting records come from continental shelf waters of the Northern Hemisphere, according to the Ocean Biogeographic Information System (OBIS, www.iobis.org, 05/2010). Unlike other species distribution models, the RES model therefore is based primarily on expert knowledge, compiled through extensive literature review, supplemented by occurrence data (where possible). This synthesized information is used to assign species to pre-defined habitat use categories, represented by simple trapezoid response curves, with respect to three basic environmental predictors [12]. For migratory species with known shifts in habitat usages during different seasons, habitat categories were selected to reflect both winter and summer usage [12]. Generic environmental predictors, including bathymetry, sea surface temperature and sea ice were selected *a priori* as predictive variables for all species, based on their documented importance in determining marine mammal occurrences directly or indirectly, e.g. through influencing prey availability. For example, strong correlations between bathymetry and patterns of species' occurrences have been noted for cetaceans and pinnipeds in different regions and ocean basins [34], [35], [36], [37]. Sea surface temperature (SST) changes may be indicative of oceanographic processes that ultimately determine predator occurrence across multiple temporal scales [38] and significant correlations of SST with marine mammal presence and species richness of different predator groups have been demonstrated across regions and taxa [e.g. 3,34]. Another key environmental parameter that has been demonstrated to determine marine mammal species presence is sea ice concentration [39], [40], since the edge of the pack ice represents an important feeding ground for many species [41]. The environmental data sets used for range predictions include gridded bathymetry data (from the ETOPO2 dataset, National Geophysical Data Center, http://www.ngdc.noaa.gov/ngdcinfo/onlineaccess.html) as well as mean SST extracted from the World Ocean Atlas [42] for the 1990s [12]; mean annual sea ice concentration data (United States National Snow & Ice Data Center (NSIDC) [43] was used instead of the formerly used data on distance to ice edge.

The RES model generates an index of species-specific relative environmental suitability of each individual half degree grid cell by scoring how well its physical attributes matched the known aspects of species' habitat use. RES values range between 0 (not suitable) to 1 (highly suitable) and represent the product of the suitability scores assigned for the individual environmental attributes (bottom depth, SST, sea ice concentration, and distance from land in some cases), which were calculated using pre-defined trapezoidal functional response curves. Model-predicted ranges and parameter settings for all species were summarized by Kaschner [44]. RES predictions for data-rich species have been successfully validated across different areas and time periods using independent data sets from dedicated marine mammal surveys [12] Validation analyses showed a strong positive relationship between the effort-corrected sighting rates of individual species and the corresponding predicted relative environmental suitability. Similarly, long-term habitat usage of species derived from effort-corrected whaling data provided support for the shape of pre-defined habitat categories used for RES input. Nevertheless, RES predictions often included parts of a species fundamental niche as well as its realized niche and suitability thresholds beyond which predicted presences were matched with observed occurrences varied by species. Since there is insufficient data to determine such presence thresholds empirically for all species, we used an alternative approach to generate species richness maps. Using a uniform presence threshold for all species, we generated richness maps across a range of different RES thresholds (RES>0 to RES = 1). To investigate how different assumptions about environmental suitability and species presence might affect predicted patterns of marine mammal richness, we then validated predictions against survey data.

### Validation with survey data

We validated our predictions of marine mammal richness using available cetacean sighting data sets collected during dedicated surveys. To avoid circularity, we only used data which had not contributed extensively to assign species to specific habitat categories in the RES model. Validation data sets included a) the IWC-IDCR circumpolar cruises conducted regularly in the Southern Ocean between 1978–2001 [45], b) four NASS surveys, conducted in 1987, 1989, 1995 and 2001 in the Northeastern Atlantic [46], and c) seven SWFSC-ETP surveys conducted across the Eastern Tropical Pacific from 1986–1990 and 1992–93 [47], [48] (Table 1). These three data sets likely represent the largest existing efforts to date to survey cetacean populations. Since pinniped observations were not reported consistently, validation analyses were limited to cetaceans only.

**Table 1:** Summary of validation data sets.

Survey Acronym	IWC-IDCR	NASS	SWFSC-ETP
**Survey Name**	International Whaling Commission - International Decade of Cetacean Research	North Atlantic Sightings Survey	Southwest Fisheries Science Centre - Eastern Tropical Pacific Surveys
**Agency/Source**	IWC Member State collaboration	North Atlantic Marine Mammal Commission (NAMMCO)	US National Marine Fisheries Service (NMFS) - SWFSC
**Time period**	1978–2001	1987, 1989, 1995 & 2001	1986–1990 and 1992–93
**Ocean basin**	Antarctica (S of 60° S)	NE Atlantic	Eastern Tropical Pacific
**No. of sighting events**	∼35000	∼7500	∼8800
**No. of identified species reported**	31	17	34

Comparison of predicted cetacean species richness from the RES model with observed richness from cetacean surveys was performed on a 5°x5° grid to ensure sufficient sightings to estimate species richness for each cell empirically. Sightings data per 5°x5° cell were combined across all years for each survey. Only records with high certainty in species identification were included. We used rarefaction to standardise for varying survey effort in different cells [49], [50]. The rarefaction model is based on the hypergeometric distribution, sampling without replacement from a parent distribution. It is widely used to compare the number of species in a collection of samples with uneven sample sizes [51]. Species richness is expressed as the expected number of species from a standardized subsample of size *n*, which is computed as
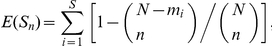
where *N* is the total number of individual sightings in the sample (here a 5°x5° cell), *S* is the total number of species in the sample, and *m_i_* is the number of individuals of species *i* in the sample. We calculated rarefied richness estimates for different *n* [52], namely the expected number of species per *n* = 20 sightings (ES_20_), 50 sightings (ES_50_) and 100 sightings (ES_100_). Selection of an appropriate *n* represents a trade-off since the range of potential diversity per sampling unit will increase with increasing *n*, but sample size (i.e. the number of cells with enough effort to produce rarefied estimates) and consequently geographic coverage will decrease.

We modeled the relationship between predicted and observed species richness using spatial eigenvector mapping (SEVM) to account for the effect of spatial autocorrelation on model results [53]. We fit Gaussian generalized linear models across all combinations of *n* and RES presence thresholds using the SEVMs package spdep v. 0.4–52 [54] in R [55] (version 2.8.1). Goodness of model fit (corrected for spatial autocorrelation) was assessed for each survey area separately as well as for all surveys combined using the coefficient of determination (adjusted r^2^), and the most parsimonious model was identified using the Akaike Information Criterion (AIC) (Table S1). To ensure the broadest geographic representation, we based the selection of a RES threshold for forward projections of species richness patterns on the model combining data from all three survey areas and including the largest possible number of cells covered by enough survey effort to be included in the rarefaction analysis. Threshold selection was thus based on lowest AIC of the combined data set model for the lowest possible rarefaction basis, but excluded all *n* values and RES threshold combinations for which models did not produce significant relationships with validation data at the level of individual surveys (Table S1).

Since the validation analysis did not allow the unequivocal identification of a single best RES threshold model across all rarefaction bases *n* and survey areas, we also calculated the variation in predicted species richness for different ranges of RES thresholds for each survey cell. Mean standard deviation and coefficients of variations computed across all cells covered by a given survey and for all surveys combined can then provide an indication of the uncertainty in predicted species richness associated with the threshold selection process (Table 2).

**Table 2:** Effects of RES threshold selection on predicted species richness.

Survey Area	Variation in predicted species richness (number of species)					
	0.00< RES ≤1.00		0.25≤ RES ≤0.75		0.55≤ RES ≤0.65	
	Mean SD	Mean CV	Mean SD	Mean CV	Mean SD	Mean CV
**SWFSC-ETP**	5.68	0.26	2.84	0.13	1.02	0.05
**NASS**	3.04	0.24	1.60	0.13	0.35	0.03
**IWC-IDCR**	3.42	0.36	1.63	0.17	0.72	0.11
**All Surveys**	3.98	0.32	1.95	0.16	0.76	0.08

Variation (expressed as standard deviation, SD and coefficient of variation, CV) in number of species predicted to occur in each surveyed 5° cells between different assumed RES thresholds, averaged across all cells covered by a given survey. Estimates correspond to the level of uncertainty associated with predicted species richness in different survey areas that is introduced by the threshold selection process to generate species richness maps.

### Forward projections

To assess potential effects of climate warming and sea ice change on marine mammal biodiversity, we projected future distributions using mean temperatures and ice concentrations derived from the IPCC climate change scenario A1B for the years 2040–2049. This ‘intermediate’ scenario assumes very rapid economic but low population growth, rapid introduction of new and more efficient technologies, and moderate use of resources with a balanced use of technologies [56]. Assuming that species would maintain the same environmental preferences with respect to SST and sea-ice concentrations, we generated predictions of future species distribution for all species and superimposed them, applying the best presence threshold as determined by our validation with survey data. To assess changes in species richness and distribution over time, we compared future patterns with current ones produced from a control data set (mean modeled 1990–99 environmental data) from the same climate scenario. Changes in species richness were shown in terms of absolute loss of native species from a given area or the absolute number of species that were newly predicted to occur in a given cell relative to the 1990–99 scenario. Similarly, we computed proportional increases and decreases in net biodiversity for each cell and the expected total and relative change in the number of species for different taxonomic groups. To assess potential effects of climate change on individual species, we also calculated the change in the size of distributions for each species between 1990–99 and 2040–49. Following the approach of a similar study [23], we then divided species into those that were predicted to expand, contract, or show no change in their range size. To provide an indication of the extent of the expected effect across different taxa, we computed the mean proportional change in size of distribution across all species falling into a specific category in each taxon.

## Results

### 1990s species richness

**Figure 1:**
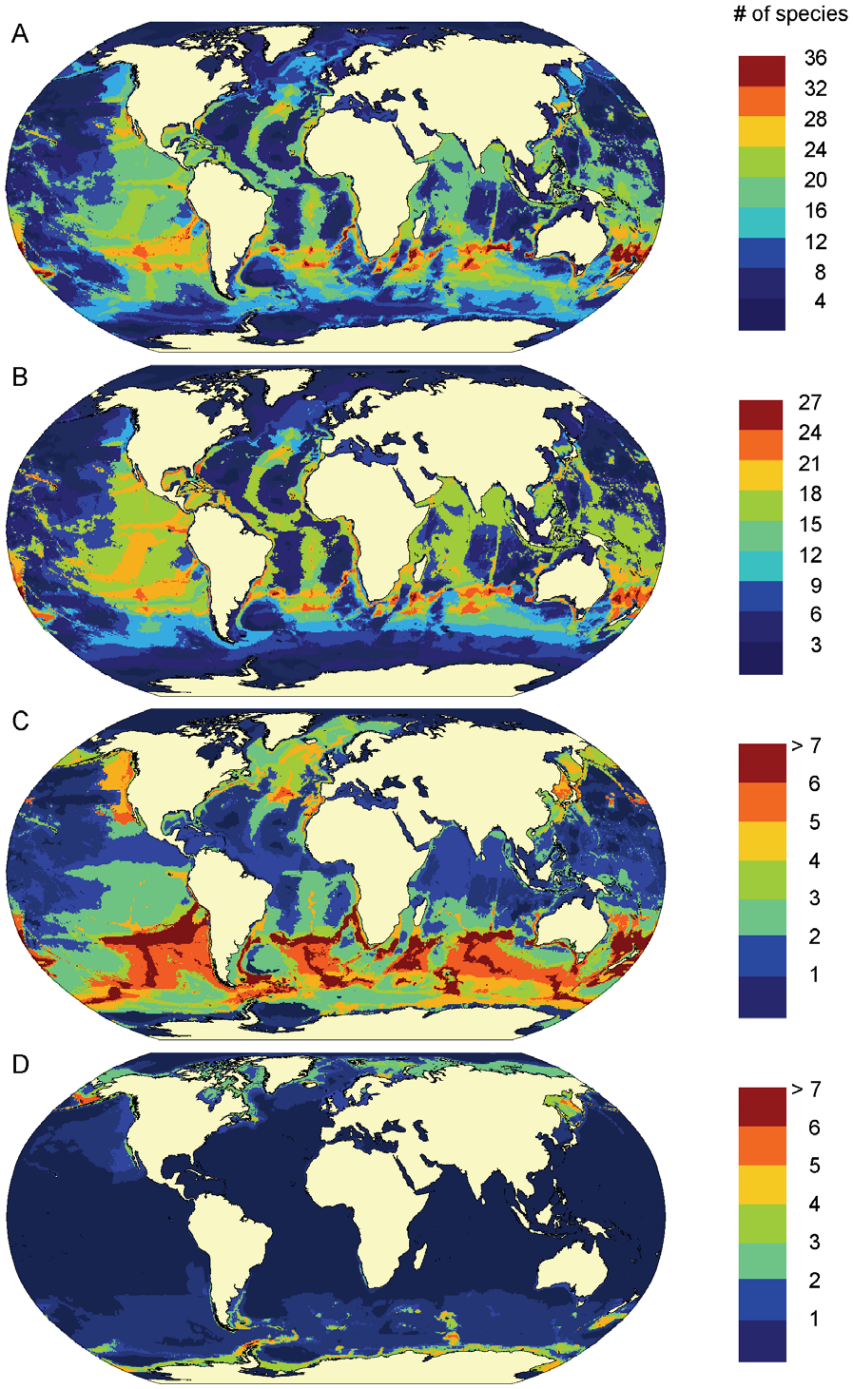
Predicted patterns of marine mammal species richness. **A.** All species (n = 115), **B.** Odontocetes (n = 69), **C.** Mysticetes (n = 14), **D.** Pinnipeds (n = 32). Colors indicate the number of species predicted to occur in each 0.5°x0.5° grid cell from a relative environmental suitability (RES) model, using environmental data from 1990–1999, and assuming a presence threshold of RES>0.6.

Predicted patterns of marine mammal biodiversity were relatively consistent across all assumed RES thresholds, showing broad bands of high species richness in temperate waters of both hemispheres (Figure S1). Patterns based on a presence threshold RES>0.6 (Fig. 1) were most strongly supported by empirical species richness data (see ‘Validation of species richness’, below). The largest concentrations of marine mammal biodiversity were found in temperate waters between 20–50°S where up to 30% of all species may co-occur (Fig. 1A). Southern-hemisphere hotspots of high species richness were predicted in waters surrounding New Zealand, some Sub-Antarctic and Southeastern Pacific islands, and offshore waters along the coasts of southern South America. Biodiversity was also predicted to be high in subtropical and temperate waters of the Northern Hemisphere, although hotspots tended to be fewer and smaller in size. These included the waters surrounding Japan and Korea, Northwest Africa, the Southeastern U.S., parts of the mid-Atlantic ridge, Baja California, the Galapagos Islands and Hawaii. Overall, hotspots were relatively small: the total area of hotspots containing more than the 75^th^ percentile of the maximum predicted species richness amounted to less than 5% of the oceans. Areas of high diversity were more abundant in the southern hemisphere where many species are more wide ranging and distributions tend to be less restricted by land barriers.

The comparison of species richness maps for different subgroups (Fig. 1B–D) with the overall species richness pattern shows that hotspots are probably mostly influenced by predicted odontocete species occurrence. Both odontocetes (Fig. 1B) and mysticetes (Fig. 1C) showed a band of high species richness in temperate waters of the Southern Hemisphere. However, while odontocete species richness was also high along ocean ridges in warmer waters (Fig. 1B), mysticetes concentrated in mid-latitudes (Fig. 1C). Distributional ranges for both groups were relatively large on average, resulting in large areas of overlap where many species co-occur. In contrast, pinniped species richness was mostly concentrated in subpolar and polar waters, and the lower degree of overlap in distribution between species resulted in ’weaker’ hotspots with only up to six co-occurring species (Fig. 1D). Pinniped hotspots were located around the Sub-Antarctic islands and the Antarctic Peninsula, in the Bering Sea and the Sea of Okhotsk (Fig. 1D).

**Figure 2:**
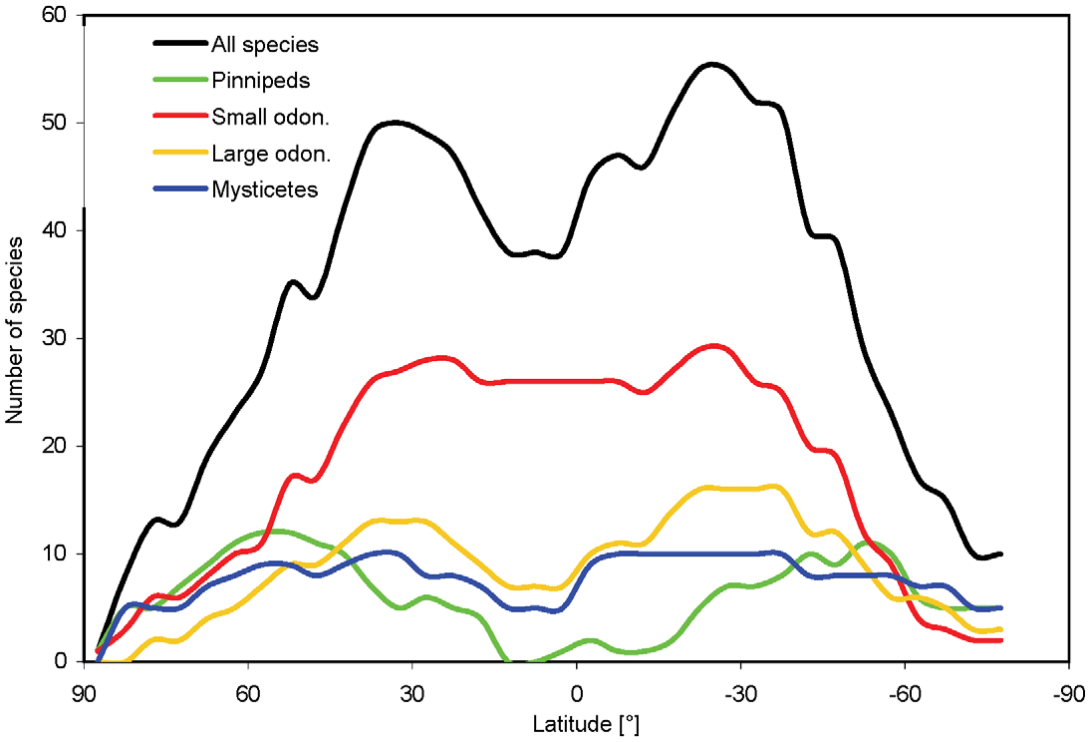
Marine mammal species richness by latitude. Number of predicted species was summed over 5° latitudinal bands for all species, mysticetes, small odontocetes, large odontocetes (beaked whales and sperm whale), and pinnipeds.

Latitudinal gradients of predicted marine mammal richness showed a bimodal distribution, with total species richness lowest in polar regions, highest between 30–60° N or S, and intermediate in tropical waters (Fig. 2). This basic pattern was shared across groups, although peaks in species richness occurred more pole-wards and tropical richness was much lower in pinnipeds compared with cetaceans. Small odontocetes (dolphins and porpoises) had the highest number of species of all groups, particularly at subtropical and tropical latitudes (Fig. 2).

### Validation of 1990s species richness

**Figure 3:**
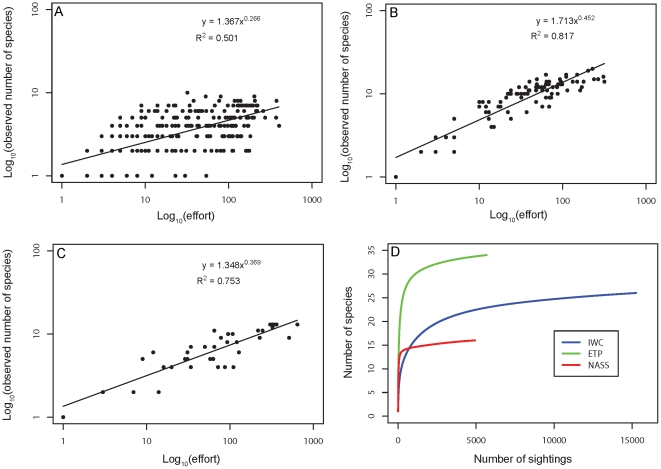
Rate of species discovery with survey effort. Number of species detected with increasing sampling effort in each 5°x5° cell in **A.** Antarctic waters, **B.** The Northeastern Atlantic, **C.** The Eastern Tropical Pacific, **D.** Species accumulation curves in different survey areas.

For all three cetacean surveys we observed a strong linear relationship between the number of species seen in a given area and effort, expressed as total number of sightings in that area (Fig. 3 A–C). This suggests that the use of rarefaction is necessary to account for uneven effort across cells. None of the rarefaction curves calculated for each survey reached a full asymptote, which suggested that surveys are still incomplete in terms of marine mammal species detection (Fig. 3D). This may in part be explained by the difficulty to distinguish some closely related species, such as the numerous *Mesoplodon* spp. (beaked whales) at sea, sightings of which are often reported at a higher taxonomic level and thus would not be considered in this analysis. Survey effort was greatest in Antarctic waters in terms of sightings, but the total number of species observed in this region was still 30% lower than in the Eastern Tropical Pacific (Fig. 3D).

**Figure 4:**
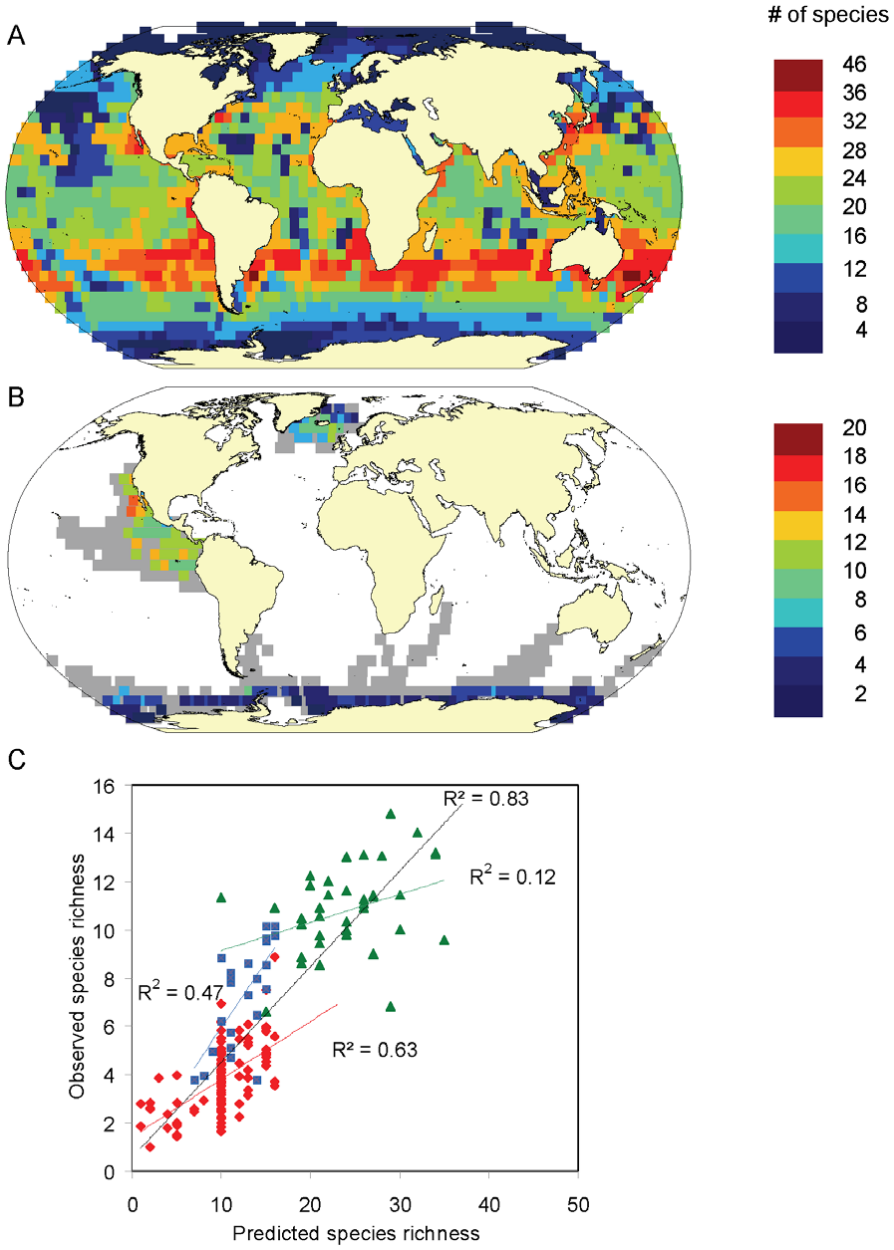
Validation with empirically observed marine mammal occurrences (5°x5° cells, 1990–1999). **A.** Predicted species richness of all cetaceans (RES presence threshold >0.6), **B.** Observed cetacean species richness per standardized sample of 50 sightings (grey cells have been covered by surveys but had insufficient effort for analysis), **C.** Relationship between observed and predicted species richness in the Antarctic (red), North Atlantic (blue) and Eastern Tropical Pacific (green) and across all three surveys (black). Data points correspond to individual 5°grid cells, regression lines to best linear fits, r^2^ values were corrected for spatial autocorrelation.

We found significant linear relationships between predicted (Fig. 4A) and observed rarefied richness (Fig. 4B) for all three survey areas individually as well as combined (Fig. 4C). Significant linear relationships were seen across a wide range of different rarefaction bases and RES presence thresholds (Table S1). Presence thresholds associated with the most parsimonious models (lowest AIC) varied among survey areas and rarefaction bases, ranging between 0.25<RES<0.75, making the selection of a single best threshold somewhat subjective. However, the variation in species richness predicted for each 5° cell across this range of RES thresholds was relatively small on average, amounting to only 16% of the total number of predicted species or ±2 species on average (Table 2). This indicates that predicted estimates of absolute species richness appear to be relatively robust across a range of thresholds. For display purposes we used the RES threshold >0.6, associated with the second lowest AIC for all surveys combined at rarefaction basis *n* = 50 (i.e. expected species per 50 sightings). This threshold was associated with the lowest possible ES basis to ensure the widest possible geographic coverage, while at the same time consistently producing significant relationships and good model fits at the level of individual surveys (Table S1). We note, that the species richness maps based on the RES>0.6 threshold correspond to areas of overlap in highly suitable habitat across many species, but species may also occur in habitat predicted to be less suitable than the selected threshold].

Based on the estimated regression slope of our best model, only between 10–50% of all species predicted to occur in a given 5°x5° cell had actually been observed in any of the survey areas given the effort of survey data sets included in the analysis (Fig. 4C).

### Forward projections

**Figure 5:**
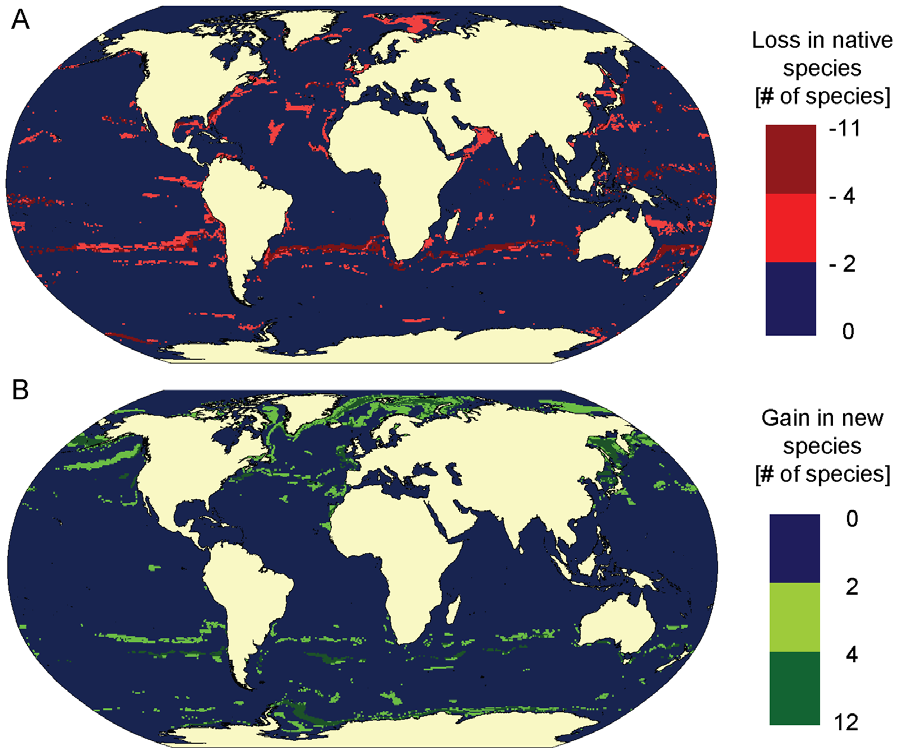
Projected effects of climate change on marine mammal species richness. Projected changes in overlap of optimal habitat across all species from 1990–1999 to 2040–2049 using the IPCC-A1B climate change scenario (0.5°×0.5° grid cells) **A.** Loss in number of native species, **B.** Gain in number of new species. Biodiversity changes are expressed relative to species richness predicted for the 1990s, and assuming a presence threshold of RES>0.6.

Projecting environmental change according to the intermediate IPCC-A1B climate change scenario for the years 2040–49, the predicted effects on global marine mammal biodiversity based on RES >0.6 were moderate (Fig. 5). Although the absolute loss in optimal habitat for native species might regionally affect as many as 11 species, this is predicted only in relatively small areas (Fig. 5A). In the Northern Hemisphere, the areas most likely to experience a decrease in the number of native species were the Barents Sea, parts of the North Atlantic ridge, and the Northern Indian Ocean as well as waters surrounding Japan (Fig. 5A). In addition, species loss was predicted to occur along coastlines or across continental shelves (Fig. 5A). In the Southern Hemisphere, decreases in native species richness were predicted mostly along 30° south, but also around the Galapagos Islands and in the Coral Triangle (Fig. 5A). At the same time, increases in biodiversity, mostly through the invasion of new species in polar waters, might also be substantial, particularly in the Northern Hemisphere (Fig. 5B). Areas most likely to experience an increase in the number of species due to invasion, were the Northern Greenland Sea, the Barents Sea, and the central Bering Sea as well the high Arctic waters (Fig. 5B), where temperature increases might enable colonization of up to 10 new species. In the Southern Hemisphere, as sea ice melts and retracts, species richness might also increase substantially in parts of the Weddell Sea (Fig. 5B). Roughly 84% of all areas in which marine mammals were predicted to occur may experience only small changes in species composition and richness due to projected changes in temperature or sea ice concentration (dark blue areas in Fig. 5A, B, where predicted changes are within the bounds of uncertainty associated with the RES threshold selection process, see above).

**Figure 6:**
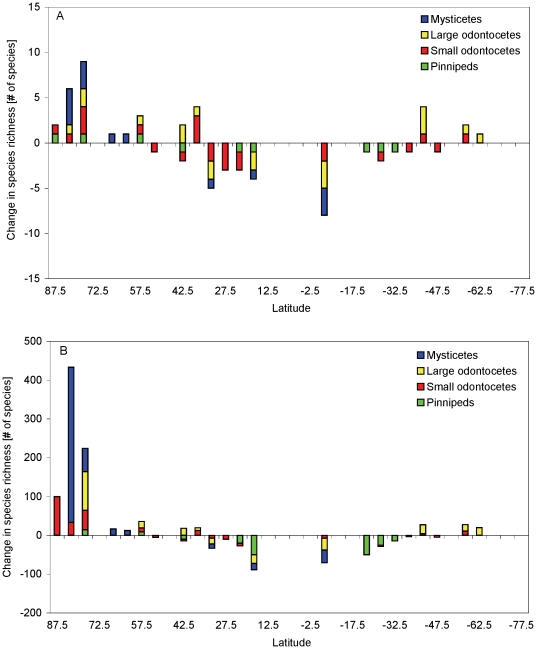
Projected absolute and proportional changes in marine mammal species richness and community composition at different latitudes. Changes were calculated relative to predicted species richness for the 1990s summed over 5° latitudinal bands for mysticetes, small odontocetes, large odontocetes, and pinnipeds.

With respect to individual taxa, pinniped biodiversity in tropical and temperate waters was predicted to decrease substantially (Fig. 6A), with the Galapagos fur seal (*Arctocephalus galapagonensis*) and the Hawaiian monk seal (*Monachus schauinslandii*) being most affected, but not the Galapagos sea lion (*Zalophus wollebaeki*). In contrast, the number of mysticete species at high latitudes, of the northern hemisphere in particular, was predicted to increase substantially (Fig. 6A). Overall, changes in species composition in terms of absolute number of species in different taxa were predicted to be highest in tropical waters, but taxonomically, the proportional composition of marine mammal communities was predicted to change most drastically in Arctic waters (Fig. 6B).

Increases in range size were predicted for 54% of all species, while 45% might experience a net loss in range size, and 1% of all species may not change (Table 3). However, these changes will typically be small, i.e. less than 10% for most taxa (Table 3). Notable exceptions include a substantial increase of predicted suitable habitat, as defined by our model, for endangered North Pacific (*Eubalena japonica*) and Atlantic (*E. glacialis*) right whales (15% and 27% increase respectively), the gray whale (*Eschrichtius robustus*) (40%) and Steller sea lions (*Eumetopias jubatus*) (85%).

**Table 3:** Predicted changes in mean size of optimal ranges due to climate change by the years 2040–49 across different taxa (IPCC-A1B scenario & RES>0.6).

		Range expansion		Range contraction		Stable range size		
Suborder	Family	Mean increase [%]	# of species	Mean decrease [%]	# of species	No change	# of species	Total
**Pinnipedia**	**Otariidae**	35.47	3	−8.82	12			15
**Pinnipedia**	**Phocidae**	28.02	11	−4.77	5			16
**Pinnipedia**	**Odobenidae**			−3.15	1			1
**Mysticeti**	**Balaenidae**	20.68	2	−3.98	2			4
**Mysticeti**	**Balaenopteridae**	1.70	3	−3.21	5			8
**Mysticeti**	**Eschrichtiidae**	40.12	1					1
**Mysticeti**	**Neobalaenidae**			−4.49	1			1
**Odontoceti**	**Delphinidae**	6.44	24	−4.55	11			35
**Odontoceti**	**Kogiidae**	0.68	1	−14.19	1			2
**Odontoceti**	**Monodontidae**	5.70	1	−8.95	1			2
**Odontoceti**	**Phocoenidae**	15.14	3	−3.20	2	0	2	7
**Odontoceti**	**Physeteridae**	0.48	1					1
**Odontoceti**	**Pontoporiidae**			−5.85	1			1
**Odontoceti**	**Ziphiidae**	4.27	12	−3.87	9			21
	**Total**		62		51		2	115

## Discussion

We used a model combining empirical observations, expert-derived range maps, and environmental niche associations to predict present-day and future distributions of marine mammals. Validation with available survey data sets indicated that broad patterns of species richness are reproduced reasonably well, and lend confidence to the global approach taken here. Forward projections based on expected changes in temperature and sea ice concentration alone suggested modest changes over the course of the next 40 years, with possible declines in marine mammal species richness at lower latitudes and increases at higher latitudes, assuming an intermediate IPCC climate change scenario.

Low spatial coverage, relative to the global distribution of marine mammals, is a problem of marine mammal surveys in general (our Fig. 4B), and shows how much is still unknown with respect to the distribution of these animals. Yet even in our best-surveyed regions, there was still evidence of an incomplete inventory of species richness (Fig. 3D). However, continuing survey efforts, such as those that have been conducted in the ETP since 1993, are expected to improve species inventories over time. Nevertheless, fully complete survey-based inventories will likely remain a challenge, given, for example, the rarity and low detectability of numerous beaked whale species, combined with difficulties to distinguish species at sea. Similarly, seasonal coverage of existing surveys rarely exceeds the summer months, and seasonal occurrences of migratory species may thus be missed, but will be included by a model that predicts long-term annual average occurrences. Consequently, the use of environmental suitability models might be viewed as a complementary tool to explore patterns of biodiversity, particularly in less well-surveyed regions around the world. Modeled species ranges can then be refined and validated as new survey data continue to be collected.

Our predictive maps show distinct peaks of marine mammal species richness in temperate waters of both hemispheres, similar to those that have been found for other marine predators and zooplankton [3], [57]. These areas represent highly productive oceanographic transition zones (e.g. [58]), where range extents of tropical and temperate species overlap. The much stronger peak in the Southern hemisphere might be explained by macro-evolutionary patterns of speciation in the absence of geographic barriers – this may have resulted in a much greater number of panglobal species in the Southern compared with the Northern hemisphere.

Our results compare well with patterns of global marine mammal diversity reported by the International Union for the Conservation of Nature (IUCN) mammal specialist group [6]. Hotspots of species richness and latitudinal patterns reported by the IUCN are similar to those reported here, although species richness in the IUCN maps appears to be higher in tropical waters than in our analysis, and the latitudinal bands of high biodiversity in temperate waters are less pronounced. Most of these differences can be explained by the higher level of spatial detail provided by the RES models in terms of the relative probabilities of occurrence of species. Our approach relaxes the assumption of equal probability of occurrence throughout the range, which is implicit in the IUCN range maps of species. This assumption effectively translates into large proportions of the oceans to be represented as almost homogenous in terms of species richness, given the high number of cosmopolitan or pantropical species. For instance, in the IUCN study [6] many of the baleen whales with maximum ranges extending from pole to pole contribute to the equatorial band in high species richness, even though the occurrence of baleen whales in tropical waters is limited to a few species, such as the Bryde's whales (*Balaenoptera brydei* and *B. edeni*), humpback whales (*Megaptera novaeangliae*) or some resident blue whale populations (*B. musculus*) [59], [60]. As a further consequence of this approach, predicted biodiversity hotspots are likely determined largely by overlapping species with restricted ranges, while possible concentrations of cosmopolitan species may be masked by the assumed uniform global occurrences. In contrast, our non-binary predictions of species-specific relative environmental suitability combined with the selected threshold of >0.6 effectively describe geographic areas of predicted co-occurrence of highly suitable habitat for many species. As a consequence RES-model derived hotspots, for instance, were more concentrated in temperate waters and around topographical features such as the mid-Atlantic ridge and seamounts [see also 61] than suggested by the IUCN maps. In the context of marine spatial planning, information on the relative importance of areas throughout a species range can help identify areas where the implementation of conservation measures will be most beneficial, to ensure the protection of both individual species and marine mammal biodiversity.

It should be noted, however, that the importance of bathymetry in determining species occurrence might be overestimated by our approach, and that observed patterns of species richness might bear less resemblance to bathymetric maps if additional environmental parameters were taken into consideration. For instance, a modified version of the RES model, the AquaMaps model (available: www.aquamaps.org, [62]), has incorporated primary production, shown to be an important driver of global species richness patterns for marine mammals [3], and salinity as additional optional environmental proxies. Resulting distributions are very similar to RES based maps, but appear less dominated by bathymetry. AquaMaps outputs have also been successfully validated for some species [63], but not all marine mammal species have been fully reviewed and the relative importance of the additional parameters still remains to be thoroughly investigated. We have therefore opted for conducting the present analysis based on RES predictions for the time being.

Another study of marine mammal biodiversity analyzed empirical sightings data for deep-water cetaceans [10]. While this approach provided important insights, the disadvantage is that only a subset of species can be included, and spatial coverage is necessarily low. Yet when we compare broad latitudinal patterns of species richness, these empirical results match well with our predictions. This previous study also supported a correlation between ocean temperature and patterns of marine mammal richness [10], an assumed driving factor in our RES models.

A frequent application of bioclimatic envelope models is to project changes in distributional ranges using modeled climate change scenarios [14], [64], [65]. Recent studies provide some support that such modeled shifts in species distribution match observed range expansions towards higher latitudes [66], [67]. The results presented here match relatively well with the broad-scale predictions derived independently by Whitehead et al. [10], who also explored various climate change scenarios, and their possible effects on deepwater cetacean richness. Our approach provides more detail, as projections are based on the ranges for individual species, rather than total species richness. This allows for differing responses among species. Furthermore we do not need to assume a consistent relationship between richness and temperature. Finally, we also included ice cover, which determines food availability and breeding habitat for many of the polar species [68], [69], and is expected to influence marine mammal species at higher latitudes [30], [70], [71], [72]. Nevertheless, predictions of latitudinal patterns in species richness agree among the two studies.

Overall, our findings provide support for hypothesized impacts of climate change on cetacean ranges based on recently developed theoretical framework [23]. Therein it was proposed that the majority of cetacean species will experience some climate-change-driven range expansion or contraction [23], which matches our results qualitatively (Table 3). Our modeling approach, however, indicates that, over the course of the next 40 years, negative effects such as net range contractions may be modest for most species, while a number of species might benefit from substantial increases in optimal habitat.

Despite the encouraging empirical validations of RES predictions [this paper and 12,63] as well as the observed agreement with findings from two independent studies [6], [10], we emphasize that there are obvious limitations to our approach. For example, assumed static habitat usage of species over time is a strong assumption in a highly dynamic marine environment. Backwards validation of predicted temporal changes using historic data sets would be one potential avenue for assessing the robustness of our predictions beyond the time period of data collection. Another limitation of this study lies in our focus on a single snapshot projection of species richness into the future. Further research using time series projections based on intermediate intervals would allow the assessment of possible effects of intermittent temperature fluctuations on species distribution that could result in local extinction of populations or species not detectable in this analysis.

Our approach also cannot reproduce the full range of factors that affect marine mammal distributions today or in the future. Most important among the variables not considered by our model are the distribution of food supply, and the availability of breeding habitat, both of which could change under various climate change scenarios [30], [70], [71], [72]. Although it has been proposed that prey distributions may also shift to higher latitudes [14], [73], there is some evidence that overall prey abundance and biomass may decline in some areas [74]. It is difficult to assess how marine mammal species, which are often opportunistic foragers, will respond to shifts or reductions in prey distributions caused by increasing temperatures, but this could have an equal or greater effect on marine mammal distributions than the direct effects of temperature modeled here. Similarly, indirect effects such as changes in species interactions [27] or population dynamics [75] cannot be captured by our approach. Finally, ocean chemistry, also changing due to the uptake of anthropogenically produced carbon dioxide [76], will potentially impact calcareous organisms, the effects of which may propagate up the food-web. The general paucity of relevant data for the majority of species will likely preclude the consideration of these more complex factors in our models for the foreseeable future.

In conclusion, our models should be interpreted as minimally realistic models that generate testable predictions about the distribution of individual species and biodiversity and how these might be impacted by climate change. These predictions must be further scrutinized with independent empirical data as they become available, in order to be useful for conservation planning or management purposes. Using forward projection, RES models can be usefully applied to investigate potential future effects of climate change, which we have illustrated here using the 2040–2049 snapshot as an arbitrary reference point. However, given that climate models predict effects of global warming to become more pronounced during the later half of the 21^st^ century, the investigation of long-term changes in marine mammal biodiversity patterns in smaller time intervals and beyond the year 2050 are needed to more comprehensively assess the effect of climate change on marine mammals. With these caveats in mind, however, we conclude that the RES model represents a powerful exploratory tool to investigate the large scale occurrences patterns of taxa for which global distributional data are still remarkably incomplete.

## Supporting Information

Figure S1 **Predicted current patterns of global marine mammal species richness based on different presence thresholds.** Relative environmental suitability (RES) threshold for assumed species presence in 0.5° grid cells (1990s). A. RES>0, B. RES>0.2, C. RES>0.4, D. RES>0.6, E. RES>0.8. Biodiversity hotspots in maps based on higher assumed RES thresholds represent areas of overlap in predicted optimal habitat of many species.
(PDF)

Table S1 **Summary of validation results comparing observed versus predicted species occurrence per 5° grid cell in different survey areas.** Red values represent models with lowest AIC, yellow values correspond to models falling into the range of ΔAIC <2 and grey cells represent models with non-significant relationships.
(PDF)
