# Supplementary figures and images for: Current and Future Patterns of Global Marine Mammal Biodiversity
Source: PLoS One. 2011 May 23;6(5):e19653. doi: 10.1371/journal.pone.0019653 (PMC3100303; doi:10.1371/journal.pone.0019653)

Figure S1.

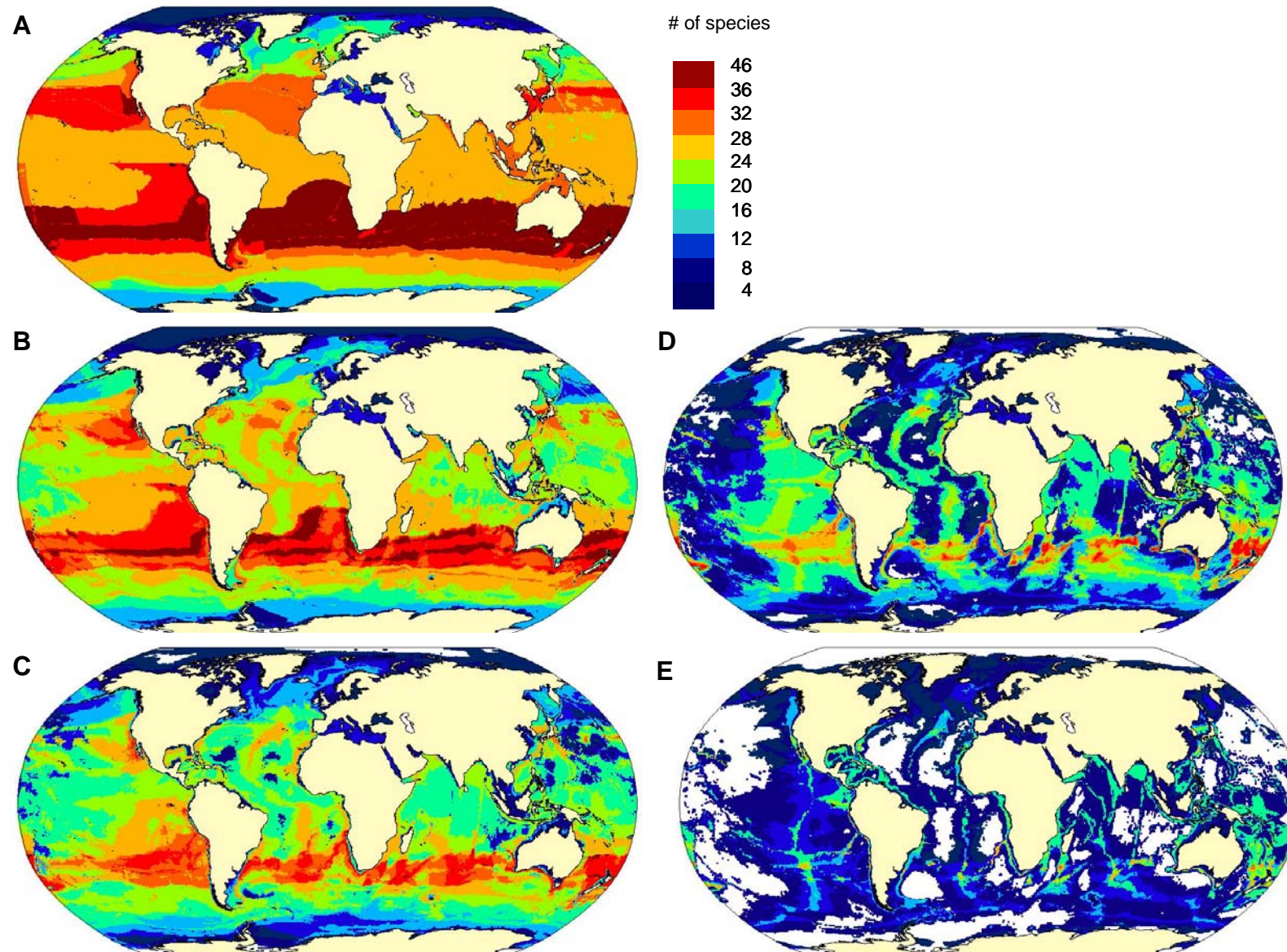

Supplement: Figure S1 — Predicted current patterns of global marine mammal species richness based on different presence thresholds. Relative environmental suitability (RES) threshold for assumed species presence in 0.5° grid cells (1990s). A. RES>0, B. RES>0.2, C. RES>0.4, D. RES>0.6, E. RES>0.8. Biodiversity hotspots in maps based on higher assumed RES thresholds represent areas of overlap in predicted optimal habitat of many species. (PDF) [file pone.0019653.s001.pdf]
