# Supplementary material for: Current and Future Patterns of Global Marine Mammal Biodiversity
Source: PLoS One. 2011 May 23;6(5):e19653. doi: 10.1371/journal.pone.0019653 (PMC3100303; doi:10.1371/journal.pone.0019653)
Supplement: Table S1 — Summary of validation results comparing observed versus predicted species occurrence per 5° grid cell in different survey areas. Red values represent models with lowest AIC, yellow values correspond to models falling into the range of ΔAIC <2 and grey cells represent models with non-significant relationships. (PDF) [file pone.0019653.s002.pdf]

Table S1

AIC

RES threshold for predicted species presence

Survey	Rarefaction basis	0.00	0.20	0.25	0.30	0.35	0.40	0.45	0.50	0.55	0.60	0.65	0.70	0.75	0.80	1.00
All surveys	20	743.17	747.23	751.35	738.47	743.09	748.42	746.56	747.63	736.46	735.96	746.32	753.80	758.94	756.48	764.29
	50	606.95	600.60	608.03	607.85	605.27	592.15	588.48	588.44	590.00	582.62	584.14	581.45	594.22	595.51	609.60
	100	353.24	325.07	314.99	318.80	318.00	318.91	318.25	323.09	322.48	324.51	345.50	348.87	351.89	340.30	382.07
IWC-IDCR	20	416.74	405.41	410.32	404.49	412.22	412.42	411.24	415.98	409.45	413.23	413.60	418.80	410.85	419.20	430.77
	50	278.96	276.46	274.39	277.91	273.30	280.24	277.63	275.31	266.35	275.53	264.99	275.33	269.03	281.49	287.41
	100	218.64	206.16	201.99	203.25	201.07	199.84	202.97	208.34	209.26	211.12	215.75	216.93	214.24	218.42	208.23
NASS	20	88.77	89.09	89.02	95.56	85.56	91.08	88.40	85.99	86.36	87.26	85.28	86.24	78.15	80.34	79.57
	50	90.06	89.12	88.73	88.59	90.68	92.26	90.06	86.35	88.41	89.76	88.23	89.63	83.31	84.08	85.57
	100	60.89	46.20	46.69	49.82	48.25	48.06	48.41	42.13	44.06	53.18	52.22	44.31	45.11	51.62	54.24
SWFSC-ETP	20	210.72	209.60	209.36	210.93	210.97	210.82	210.91	210.97	210.97	210.60	210.95	210.31	216.99	210.80	210.68
	50	161.07	162.16	162.36	159.86	159.48	160.03	160.15	159.65	160.12	160.42	161.98	163.22	163.60	164.30	165.60
	100	60.94	57.45	56.77	53.16	56.26	53.96	58.23	57.45	53.00	48.68	53.46	52.17	55.30	51.75	60.15

adjusted R^2

RES threshold for predicted species presence

Survey	Rarefaction basis	0.00	0.20	0.25	0.30	0.35	0.40	0.45	0.50	0.55	0.60	0.65	0.70	0.75	0.80	1.00
All surveys	20	0.79	0.78	0.78	0.79	0.79	0.78	0.80	0.79	0.79	0.79	0.79	0.78	0.78	0.78	0.77
	50	0.80	0.81	0.80	0.80	0.80	0.82	0.82	0.82	0.82	0.83	0.83	0.83	0.83	0.82	0.81
	100	0.85	0.88	0.90	0.89	0.89	0.89	0.89	0.89	0.89	0.88	0.86	0.85	0.85	0.87	0.80
IWC-IDCR	20	0.38	0.43	0.42	0.44	0.40	0.40	0.40	0.38	0.41	0.39	0.39	0.37	0.41	0.37	0.33
	50	0.62	0.63	0.64	0.62	0.64	0.61	0.62	0.63	0.66	0.63	0.67	0.62	0.65	0.59	0.58
	100	0.38	0.48	0.51	0.50	0.52	0.52	0.49	0.46	0.43	0.42	0.37	0.36	0.39	0.37	0.49
NASS	20	0.44	0.43	0.43	0.24	0.50	0.35	0.41	0.45	0.45	0.43	0.47	0.45	0.58	0.55	0.56
	50	0.51	0.53	0.54	0.54	0.50	0.41	0.46	0.54	0.50	0.47	0.51	0.47	0.60	0.59	0.56
	100	0.38	0.81	0.80	0.72	0.75	0.75	0.74	0.84	0.81	0.64	0.66	0.83	0.83	0.72	0.66
SWFSC-ETP	20	0.14	0.16	0.16	0.14	0.14	0.14	0.14	0.14	0.14	0.15	0.14	0.15	0.03	0.14	0.15
	50	0.11	0.08	0.08	0.13	0.14	0.13	0.13	0.14	0.13	0.12	0.09	0.06	0.05	0.03	0.00
	100	0.49	0.59	0.61	0.73	0.62	0.72	0.62	0.64	0.74	0.80	0.69	0.75	0.65	0.76	0.57
